# What do older adults think about when formulating implementation intentions for physical activity? Evidence from a qualitative study

**DOI:** 10.1111/bjhp.12621

**Published:** 2022-08-24

**Authors:** Valérie Désirée Bösch, Lisa Marie Warner, Samuel R. Nyman, Julius Haftenberger, Kye Clarke, Jennifer Inauen

**Affiliations:** ^1^ Department of Health Psychology and Behavioral Medicine University of Bern Bern Switzerland; ^2^ Department of Psychology MSB Medical School Berlin Berlin Germany; ^3^ Bournemouth University Clinical Research Unit, Department of Medical Science & Public Health Bournemouth University Bournemouth UK; ^4^ Department of Psychology Bournemouth University Poole UK

**Keywords:** beliefs, implementation intentions, older adults, physical activity, qualitative, think aloud

## Abstract

**Objectives:**

Physical activity is an important health behaviour especially for older adults. Forming implementation intentions is an effective strategy to implement physical activity in daily life for young and middle‐aged adults. However, evidence for older adults is inconclusive. This study explored the thoughts of older adults about implementation intentions and potential barriers and facilitators while formulating them.

**Methods:**

Three samples of older adults from the United Kingdom (*n* = 8), Germany (*n* = 9) and Switzerland (*n* = 17) were prompted to think aloud while formulating implementation intentions to be more physically active. After the task, semi‐structured interviews were conducted. Data were analysed thematically.

**Results:**

Participants expressed pre‐established thoughts about implementation intentions (e.g. they feel too restrictive). During the formulation of implementation intentions, several barriers to creating them were reported (e.g. problems with finding cues due to absence of recurring daily routines), but participants also mentioned that forming implementation intentions acted as a facilitator for physical activity (e.g. cues as useful reminders to be active, task itself triggering self‐reflection about physical activity). After the task, participants reflected on circumstances that decrease the likelihood of enacting implementation intentions (e.g. spontaneous alternative activities, weather, health‐related barriers, Covid‐19‐related barriers), which triggered spontaneous coping planning.

**Conclusions:**

The results on barriers and facilitators of implementation intentions and physical activity from older adults' perspectives provide starting points for improving instructions for older adults on how to create implementation intentions for physical activity. Future studies are needed to investigate whether the findings extend to implementation intentions for other behaviours.

**Statement of Contribution**

***What is already known on this subject?***

- Physical activity is an important health behaviour especially for older adults.
- Implementation intentions can effectively promote physical activity in the general population.
- Some studies suggest that implementation intentions are not useful or even detrimental for older adults' physical activity.

***What does this study add?***

- In‐depth understanding of older adults' thinking process before, during and after formulating implementation intentions.
- Barriers to implementation intentions were identified, including restrictiveness of planning and difficulty to identify cues.
- Implementation intentions prompted self‐reflection, resumption of past routines and coping planning.

## BACKGROUND

Physical activity is an important health behaviour since it is accompanied by many health benefits (Warburton & Bredin, 2017). It has been shown to effectively prevent a multitude of diseases, and notably non‐communicable diseases like heart disease and diabetes (Cunningham et al., 2020). Older adults especially benefit from regular physical activity regardless of their health status (Hupin et al., 2015). However, many older adults fail to attain the World Health Organization recommendations to be moderately physically active for at least twenty to forty minutes per day (Bull et al., 2020). To increase physical activity, a variety of behaviour change strategies have been devised, of which implementation intentions have been considered to be particularly effective (Gollwitzer & Sheeran, 2006). However, evidence of their efficacy for older adults has been mixed.

### Implementation intentions

Implementation intentions are a specific planning strategy (Hagger et al., 2016; Hagger & Luszczynska, 2014), where the target behaviour is linked to a certain cue, like a certain time of day or a situation using an if‐then formulation (Gollwitzer, 1993). Alternatively, action planning encompasses different information on future physical activities, such as time, place and duration. Implementation intentions have therefore been defined as a specific way of making plans, while action planning can be considered the broader term (Hagger & Luszczynska, 2014). Forming an implementation intention facilitates recalling the target behaviour upon cue encounter, resulting in a greater likelihood that the target behaviour is executed (Gollwitzer, 1993). As an example, someone could define the implementation intention: ‘If I have eaten breakfast, then I will go for a twenty‐minute walk’. Formulating implementation intentions has been found to be an effective strategy to recognize and act upon pre‐planned opportunities to carry out a certain behaviour (Gollwitzer & Sheeran, 2006), that is to bridge the intention–behaviour gap (Sniehotta et al., 2005).

### Implementation intentions to promote older adults' physical activity

Although implementation intentions have generally been found to be a successful behaviour change strategy, their effectiveness can vary for older adults. Some studies have found implementation intentions to be effective in promoting older adults' physical activity (e.g. in older women, older adults with obesity and orthopaedic rehabilitation outpatients; Bélanger‐Gravel et al., 2013; Hall et al., 2014; Ziegelmann et al., 2006). However, a systematic review by French et al. (2014) showed that including action planning or coping planning in interventions for older adults *negatively* affected their physical activity behaviour and self‐efficacy. While implementation intentions were not included in this review, the results on very similar self‐regulatory behaviour change techniques questions the effectiveness of implementation intentions for older adults. French et al. (2014) assumed that planning in general might either be more cognitively challenging with increasing age, or less needed (e.g. due to more flexible schedules) and therefore less acceptable for older adults. Quantitative and qualitative research highlights that older adults – especially retirees – indeed report different *preferences* when forming implementation intentions. They prefer slower paced activities, more frequent activity bouts and more flexible time points instead of setting particular starting times (Alley et al., 2018; French et al., 2021). Research into the most *effective* characteristics of implementation intentions formed in a physical activity trial for older adults suggests that using daily routines as if‐cues (rather than exact times) and forming heterogeneous implementation intentions with diverse physical activities resulted in higher plan enactment (Warner et al., 2021). However, these conclusions are based on an overall non‐effective physical activity randomized controlled trial among older adults (Warner et al., 2016). Overall, these mixed findings on the effectiveness of implementation intentions in older adults, therefore calls for a deeper investigation into what exactly older adults think of implementation intentions while asked to formulate them as a specific planning strategy to increase their physical activity.

### Purpose of the present study

The aim of this study was to enhance our understanding of older adults' thought processes during the creation of implementation intentions for physical activity. We aimed to answer the following research questions: What do older adults think about when they create implementation intentions for physical activity? What barriers and facilitators do older adults experience when formulating implementation intentions?

## METHOD

### Design

We adopted a qualitative approach and used a think‐aloud paradigm (Genest & Turk, 1981). The goal of this method was to gain insight into the ongoing thought processes while formulating implementation intentions (Van Someren et al., 1994). To obtain the views of older people from a range of geographical and cultural contexts, we collected data from the United Kingdom (UK), Germany (DE) and Switzerland (CH). Ethical approval was obtained by each of the three Research Ethics Committees of the part‐taking institutions (approval numbers blinded for review).

### Participants

The study population consisted of community‐dwelling older adults aged 65+ years, sampled from the United Kingdom (*n* = 8; *M*_age_ = 72.0 years; *SD*_age_ = 6.1; data collected in September 2019–March 2020), DE (*n* = 9; *M*_age_ = 74.8; *SD*_age_ = 6.6; data collected in July 2020) and CH (*n* = 17; *M*_age_ = 74.8; *SD*_age_ = 6.6; data collected from September 2019–February 2020). Inclusion criteria in all samples were as follows: participants were community‐dwelling; able to independently participate in the study; deemed themselves capable to be physically active, which they had to assess for themselves prior to participating (in the informed consent:"If you want to partake, it is required that you can be physically active, and give your informed consent to participate"); and had not received any contraindications from a health practitioner for being physically active prior to the study. No adults with dementia were recruited, and in the Swiss sample, dementia was an explicit exclusion criterion. Participants were recruited via convenience sampling through local organizations (e.g. University of the Third Age). Additionally, snowball sampling through friends, family and neighbours was utilized. Further sociodemographic characteristics of the samples can be found in Table 1.

**TABLE 1:** Sociodemographic characteristics of participants

Sample	UK (*N* = 8)	DE (*N* = 9)	CH (*N* = 17)
Gender			
Female	3	6	7
Male	5	2	10
Marital Status			
Single	1	0	2
Married/with Partner	4	3	10
Divorced	2	1	4
Widowed	1	3	2
Highest Education			
Not specified	1	1	0
Other professional	2	2	1
A‐Levels	1	0	0
College	1	0	0
Apprenticeship	1	0	3
Secondary School	0	0	2
University	0	6	11
Retirement status			
Retired	6	8	14
Not retired	2	1	3

### Procedure

Before the interview, participants in the UK sample were provided with a participant information sheet. They were asked to discuss the information sheet and raise any potential questions prior to the interview appointment. On the day of the appointment, these questions were discussed prior to the beginning of the task and interview. Participants then completed an informed consent form. The interview started with a practice ‘think aloud’ task. Participants were asked to speak aloud about what they read, thought, and wrote. Following this, the interview was audio‐recorded and participants were asked to ‘think aloud’ during the whole interview. This enabled capturing their beliefs about implementation intentions before, during and after the task. They first read a form on the physical activity recommendations according to the World Health Organization to provide a reference point for sufficient health‐relevant moderate or vigorous physical activity (Bull et al., 2020). As most participants were active already but most not active enough in terms of the WHO recommendations, defining a healthy amount of activity was intended to motivate participants to strive for more physical activity in their implementation intentions.

They were then provided with prompts and instructions on how to create implementation intentions and asked what physical activities they would like to carry out, which ones they could do daily, when they have time each day to be active, how long they could be active each time and if they would like to be active alone or together with others. We required the implementation intentions to be formed for a daily activity as this procedure enables better habit formation with repeated cue‐action links (Lally & Gardner, 2013; also see Supplements A1 and A2; all Supplements are stored in the Open Science Framework; https://osf.io/gu9d8/?view_only=2a19fddc8d574a7caa0dd9bdd8318680). Afterwards, they were handed a planning sheet and asked to formulate up to three implementation intentions being moderately to vigorously physical active in if‐then format (see Supplement A1 and A2).

Following this, participants were questioned on their experiences with the task via a semi‐structured interview, using pre‐formulated prompts if needed. These open‐ended questions aimed to cue participants' thoughts on physical activity, completing the planning task and creating their if‐then plans (e.g. What was it like to complete the task, which aspects were motivating and if it did feel natural or not; see Supplement B). Additionally, seven demographic questions were asked (see Supplement A1 and A2). All participants were interviewed in their homes or other venues if preferred (e.g. campus, public café).

The data collection in the DE‐sample followed a nearly identical procedure. However, due to the restrictions caused by the Covid‐19‐pandemic all interviews were held online via a video‐telephony provider. Moreover, the sheets where the participants could note their implementation intentions (see Supplement A1 and A2) were sent to the participants' addresses and unpacked from a letter, when prompted by the interviewer.

The data collection in the CH‐sample was conducted within a complex intervention study to promote physical activity in community‐dwelling older adults using implementation intentions and motivational messages (blinded for review: pre‐registration and study protocol; https://osf.io/e37bn/?view_only=25386b92cefb4c25a9625ccc445c9).

The formulation of the implementation intentions was audio‐recorded with prior consent of the participants. In contrast to the DE and UK‐sample, the Swiss participants were not explicitly instructed to think aloud and had only to formulate up to two implementation intentions, but they answered the same guiding questions (see Supplement A3).

### Data analysis

**FIGURE 1:**
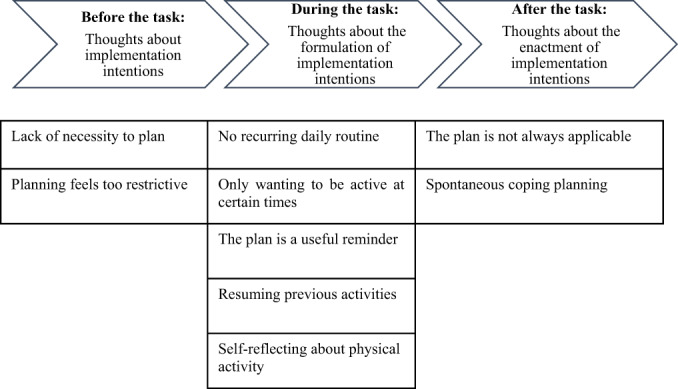
Summary of the beliefs of older adults about implementation intentions

The fourth and fifth author, and a research assistant (CH sample), transcribed the interview recordings verbatim. The data were then analysed bottom‐up by the first author, following the 6‐step thematic analysis by Braun and Clarke (2006). In the beginning, they read all transcripts, for each sample separately, to familiarize themselves with the data and then proceeded to write down initial topics. Secondly, they coded statements line‐by‐line, allowing for inclusive codes derived from the data (i.e. manifest codes), while making reflexive notes where necessary. Thirdly, initial codes were coalesced to identify relationships between the codes and combined to larger themes in each respective sample. In a fourth step, the first author reviewed the themes and compared them between the three samples. Fifth, the first and last author critically reviewed the themes and codes until they reached consensus. In a sixth step, during report writing, the first and last authors decided to order the themes along their appearance in the planning process (i.e. before, during and after the implementation intention task, see Figure 1). Then, the co‐authors reviewed a first report of the results and discussed feedback considering the research questions and the reliability of the findings. Lastly, the first author reread all the transcripts, actively searching for negative or contrasting statements to the elaborated themes. None were found.

## RESULTS

In the following, the results are presented along the temporal structure of the planning process (Figure 1). An abbreviation (UK, DE, CH) and a number indicate which sample and participant the quotations originate from (e.g. UK5 would be participant five from the UK‐sample).

### Before task: Thoughts about implementation intentions

Before writing down their personal implementation intentions, participants mentioned initial thoughts about planning that seemed to shape their attitude towards the task and its effectiveness to facilitate physical activity. This manifested in thoughts that implementation intentions are not necessary to be more active or that planning did not allow for spontaneity, and therefore was too restrictive. These themes will be further elaborated in the next paragraphs.

#### Lack of necessity to plan

A theme identified in all three samples was that planning physical activity was not necessary, because participants felt that they were already physically active on a daily basis. In those cases where participants had pre‐established routines, they saw no need in planning, as illustrated in the following statement by participant DE7: ‘I usually let things come to me and if there's time, I do it, but since everything already developed into a certain routine, I don't need to do much planning’. The formation of this belief may be due to having an established routine as seen in participant UK2, who stated: ‘I do skiing in the winter, which I've done for the last 50 years’. Another reason might be a lack of motivation, as stated by participant UK5: ‘I don't plan, yeah, I just do it when I want to do it’. These beliefs about being sufficiently physically active that can inhibit planning behaviour are best summarized by the statement of participant DE4: ‘I understood it, but honestly, I don't know what I […] could do better, what I'm already doing. I'm doing a lot already’. Overall, these participants felt that a plan was not necessary since they felt that they did not need a reminder to increase their physical activity.

#### Planning feels too restrictive

Another theme was the experience of if‐then planning as too restrictive of personal freedom. For example, Participant UK2: ‘Yeah again, physical activity ain't really something I put my mind to doing at a certain time or something, just happens so putting time to do a specific thing is kinda limiting’. Participant CH6 moreover stated that having to plan was a restraint to them: ‘Exactly, because afterwards this is somehow a restraint’. Notable here is that none of the participants stated that their belief was due to an objective barrier (e.g. pain when being active or bad weather), suggesting that this belief was a personal opinion. This can also be seen in DE7’s statement, who said that planning was too restrictive and that they therefore had trouble with the task: ‘So, I think rather restraining… like I said… I'm actually not very much of a planner and for such things… I have more of a general concept, I want to exercise as much as possible… and at which time of day or which day or so… that is a bit difficult for me to narrow it down’.

### During the task: Thoughts about the formulation of the implementation intentions

In this phase participants stated what they experienced while formulating the implementation intention. These included barriers they faced, like having no recurring daily routines, which made finding a good implementation intention difficult. In contrast, facilitators for the usage of implementations as a planning strategy were mentioned, for example that many only wanted to be active at certain times, that the plan was a useful reminder. Further themes that emerged during the task were that participants chose resuming previous activities, and engaged in self‐reflection about their current physical activities.

#### No recurring daily routine

A lack of daily routine was identified as one difficulty when formulating implementation intentions. Participant CH3, for example mentioned that they had time to be active at all times of day since they had no specific daily routine and needed help from the interviewer in finding a recurring cue for the if‐part of the implementation intention: ‘So, the only fixed routine we have is actually in the morning. Yes, early in the morning […] we get up at seven at the latest, we […] have breakfast, so actually one could only integrate something after the breakfast’. A similar scenario was mentioned by participant CH11 by stating a lack of situational cues: ‘So, a fixed thing (routine)… So, with the exception of getting up, going to bed, eating reasonably and such…, but else, in between (I have none)’.

#### Only wanting to be active at certain times

A further theme identified was knowing exactly at which times of day participants were (un)willing to be physically active. Participant CH7, for example knew exactly that they did not want to be active in the evening as this would be too strenuous: ‘[…] it would simply have to be after lunch. In the evening I would rather not do such strenuous things’. Analogously, participant CH6 said that they were not fond of being active in the morning: ‘[…] in the morning I somehow don't manage it, and after lunch I don't want at all, but before dinner it is somehow easier, and afterwards I can watch television or so’. There seemed to be a preference for activities at certain times (like in the morning or before noon). Whereas, Participant CH11 said that their favourite time to be physically active was after breakfast since this would be the only time where they were motivated to do so. They stated the reason why by saying: ‘Because, when I do it in the morning, before noon after breakfast, then firstly I have done it, secondly, I'm the most motivated, thirdly nobody disrupts me’. The activity was also frequently planned around a meal like breakfast or lunch, possibly in lack of further recurring daily cues.

#### The plan is a useful reminder

Statements regarding the if‐part of the planning task suggested that writing cues down can be a good reminder to be active. Evidence for this theme was especially strong in the UK‐sample. Participant UK1, for example, stated: ‘Usually the fact that I've written something down means that I'm more likely to do it’. In addition, the reminder was also considered as motivating as suggested by participant UK 3: ‘Well, I think writing down the plans was motivating as it gives you something to work on, like a to‐do list’. This belief was shared by UK6: ‘Again, it's a good way to remember to do physical activity I'm sure’. Interestingly, the implementation intention task encouraged mainly participants of the UK sample to think about their current physical activity.

#### Choosing to resume previous activities

Most participants across all samples had a distinct idea of the type of physical activity they wanted to perform. A minority of participants specifically chose to resume discontinued behaviours like using the stepper (a training device resembling a cross‐trainer), restarting a daily fitness routine prescribed from rehabilitation, or daily morning gymnastics. For example, participant CH4 stated: ‘So, on the one hand, what I would like to reinstate, I had done this for many years, is simply to do such a 10‐minute morning gymnastics each morning’. They moreover stated that they already knew in detail when, where and in which situation they wanted to reinstate their routine since they had done morning gymnastics for many years but had somehow stopped.

#### Self‐reflecting about physical activity

Another theme was that some participants started thinking about their physical activity in more detail. This was particularly observed in the UK‐sample. For example, participant UK6 said that they did not think enough about their activity and that the WHO recommendations made them realize that they should plan it more: ‘My first impression is probably that I don't think enough about my physical activity plans and that maybe I should devote a percentage of time to planning a bit better’. However, this could be rebutted by participant UK3 stating that they found the task ‘a bit depressing initially’ as ‘it makes you realise what I do, I should do more of’. But despite the minor negative emotional response, participant UK3 stated that the WHO recommendation and subsequent planning during the task regarding changing their amount of physical activity motivated them to be more active in the future: ‘Well it makes me feel that I want to do more’. Also, the combination of the recommendation and the task led to reflection of their current activity mainly in the UK‐sample.

### After the task: Thoughts about the enactment of implementation intentions

After completing the task, some participants remarked may face barriers while enacting their implementation intentions making its enactment not always applicable. Moreover, these barriers elicited spontaneous coping plans, foremost in the CH‐sample.

#### The plan is not always applicable

Participants noted that their formulated implementations intentions may not are always applicable and barriers for enactment of their implementation intentions were mentioned like spontaneous alternative activities, weather, health‐related problems or other appointments.

##### Spontaneous alternative activities

Most participants across the samples mentioned spontaneous other commitments as a barrier when having to implement the task. As suggested by participant UK2, prior commitments may influence the likelihood of implementing the plan, in addition to leading to prioritization of the commitment over the planned behaviour: ‘Might clash with something else I've got planned that day’. On the other hand, participant CH5 stated that they worked a part‐time job and therefore could not guarantee to implement the plan every day to the same degree, as it might coincide with their work schedule. Participant CH13 stated that they stayed in their holiday apartment for some time and therefore, could not go to the fitness studio: ‘So, within the next four weeks, I would, provided I'm not in the holiday home, surely go to the gym once’. Further, spontaneous visits from friends and family were mentioned to interfere with the plans. Participant CH16 for example stated after the question, if the plan was possible to be enacted daily: ‘Yes, sure I can. Yes, but precisely, it is just, then some visitors are suddenly coming, and then I can't go (laughs)’. A similar statement about spontaneous changes in plan was also made by participant CH12: ‘Yes, that is quite difficult then, of course. When one gets a visitor, just like me this week, […] well, then it's just difficult, that I do it then, on top’.

##### Weather

Another barrier named was weather conditions like summer heat or rainfall. Many participants stated that the weather condition was important for planning their physical activity. They remarked that implementation intentions could not always be enacted depending on the weather and should therefore be at least adaptable depending on the weather or season. For example, participant CH1 stated that they did not go walking in the afternoon in the summer due to the heat: ‘So, in summer one went in the early morning, […] as we couldn't go walking in the afternoon, […]’. Additionally, participant DE9 stated that they planned depending on the weather: ‘Ah, I plan for that, depending, on the weather and what I have planned’.

##### Health‐related barriers

Another theme comprised the belief that health could interfere with their intended implementation intentions. Participant DE9, for example stated that being sick, could lead to them not being active: ‘It might occur on a very rare occasion that I have a cold. Then I do nothing, because I know, that isn't good for anybody. But it is very, very rare that I have a cold, long time ago. And so, I have no problems there. I don't need no make a plan’. Moreover, participant UK6 answered the question of if anything could make it difficult to enact the if‐then plans with: ‘Well […], if my knee hurt or if I needed to get home quicker, I'd probably skip walking back’.

##### COVID‐19‐related barriers

Since the German sample was interviewed during the first wave of the Covid‐19 pandemic, some participants said that their established physical activity routine or plans to be physically active got interrupted. Participant DE5, for example stated that they could not go swimming now: ‘[…], but now the indoor swimming pools and everything are closed. We are of course considering, when many places are opened up again, to get an authorization online, and then also to go to the public beach or else. But we don't know yet’. Similarly, participant DE6 could not visit the gym during shutdown like they did before, which they stated inhibited them: ‘[…] in non‐Corona times I actually go four hours a week to the gym, on average, and do courses there and now that this isn't possible, I ride my bike to work, which is nearly ten kilometres’. In conclusion, seven out of nine participants reported that they somewhat faced constraints in their choice of physical activities due to Covid‐19, which manifested when having to formulate an implementation intention.

#### Spontaneous coping planning

In the CH‐sample, the instructions were to develop one to two implementation intentions, which could be implemented daily during the course of the intervention. Interestingly, a majority of the older adults in this sample automatically thought about situations in which the attainment of the plan could be more difficult like a scheduled doctor's appointment, work obligations or holidays and spontaneously formulated elaborate coping plans, even though the study design did not prompt them to do this. Participant CH5, for example, knew that they were invited to a congress for three days. Without being prompted, they thought about how they could still enact their plan of walking daily and came up with the coping plan to walk to the congress location instead of taking the bus: ‘[…] when I go to Lucerne on Wednesday and Thursday, then I will walk to the train station and also back again’. Participant CH6 also wanted to implement daily walking in the morning after breakfast. However, they automatically recalled that they had other commitments. Therefore, they stated that they would first finish these tasks and then take their daily walk: ‘Because, it is not always clear, that I can go right after breakfast, because I also have some miscellaneous appointments, […], that I have to go to’. Yet another participant, CH13, had a trip scheduled and knew that they would likely be more inclined to go skiing. On days on which they would not go skiing they scheduled a walk to the village to compensate skiing.

## DISCUSSION

The aim of the study was to determine older adults' beliefs about implementation intentions as a technique for increasing physical activity. Before the task of forming the implementation intentions, two kinds of beliefs about implementation intentions were reported. Firstly, a lack of necessity to plan, secondly that the planning feels too restrictive. Then, during the task, several barriers and facilitators were mentioned, for example that participants had no recurring daily routine, only wanted to be physically active at certain times, but also that the task was a useful reminder and encouraged resuming previous activities, and that thinking about physical activity and the planning of it triggered self‐reflection about their own current physical activity. Lastly, after the task, participants noted that the implementation intentions were not always applicable and, in some participants, the barriers elaborated in the task even triggered coping planning.

Two themes were identified that might explain why implementation intentions may fail to increase physical activity for older adults in a majority of studies (French et al., 2014). These are related to unfavourable attitudes towards implementation intentions even before starting the task, such as having the pre‐established belief that implementation intentions are either too restrictive or that it feels unnatural to formulate them, which could also be observed among younger samples (Palsola et al., 2020). This finding is in line with self‐determination theory (Ryan et al., 2009), where perceived autonomy is an important indicator for whether a person is physically active and was also found in older adults in particular (Arnautovska et al., 2018). Consequentially, implementation intentions in their strict if‐then‐form could be perceived as too restricting and therefore fail to be perceived as useful or are even opposed to by some older adults. This form of reactance was also found when asking young adults to change their diet or drinking behaviour (Sieverding et al., 2019; Ungar et al., 2015).

A further theme was the failure to understand the necessity of formulating implementation intentions, based on the belief of being active enough already without having to plan. This aligns with findings from the qualitative study from Palsola et al. (2020) where participants stated that they saw no need for planning since they already had routines for physical activity. These results depict that strong habits have to be considered in interventions and can limit the usefulness of implementation intentions, due to conscious self‐regulatory effort only being needed in early stages of behaviour change, as more automatic processes take over once a habit has been established (Di Maio et al., 2021; Gardner & Lally, 2018; Labudek et al., 2021). However, it has to be noted that strong habits may not equal sufficient physical activity: Although participants claim to be active, this could be based on a biased sense of accomplishment (Labudek et al., 2021). Yet, it is also possible that this effect arises from reading the WHO recommendations, as some participants mentioned that they were already meeting the criteria after reading them and therefore did not see the need to plan further physical activities.

However, most problems occurred during the if‐then task itself. For instance, when having to formulate the if‐part of the implementation intention, some participants deemed finding a cue for physical activity as difficult. One reason was that due to their retirement, some older adults had no event that occurred daily at approximately the same time. In these cases, they needed help from the interviewer. This finding could be based on older adults' lower prospective memory capacity for time‐based compared to event‐based cues found in laboratory studies (Henry et al., 2004). So, a practical implication could be to guide adults to plan tasks after events (e.g. meals, daily chores) rather than at particular times of day. This could also be beneficial for habit development since having a regular routine can increase the strength of repeated cue‐action links and with that automaticity of daily physical activity behaviour (Gardner & Lally, 2018).

Others also wanted to be active at specific times of the day, limiting the choice of a suitable cue. When both attitudes of having no recurring cue and only wanting to be active at certain times coincide, the selection of a suitable daily cue might be difficult, making the task less applicable for these older adults. However, this theme can also be a facilitator since participants were quickly able to identify when they would not want to be active, and so can avoid those times and plan to be active at different times. Therefore, future research is needed on whether wanting to be active at certain times is a barrier or facilitator for certain older adults.

The then‐part also identified as a useful reminder to be active. Furthermore, positive feedback was provided for the then‐part of the implementation intentions in combination with the WHO‐recommendations. Most prominently, a selection of older adults in the UK‐sample stated that this part combined with the WHO recommendations made them think more about their current physical activity. The recommendations could have triggered self‐reflection of their current physical activity levels in more detail than usually, and the task the process planning activities in general. Having a clear goal like these recommendations could enhance the effectiveness of implementation intentions on behaviour change since it is dependent on whether a person is actually motivated to perform the behaviour in the first place (Prestwich & Kellar, 2014). By reflecting on their current physical activity compared to the recommended amount during a planning intervention, older adults could become more motivated to change, rendering planning not only a volitional but also a motivational behaviour change technique to some degree. As a caveat of this study, this effect on self‐reflection is strongly linked to the WHO‐recommendations is also substantially due to reading the physical activity recommendations prior to forming implementation intentions (as they were from a credible source).

A selection of participants also stated that if‐then planning encouraged them to resume activities that they had lost track of, supporting the assumption that thinking about their current behaviour and lost routines is motivating. The task of reading the WHO recommendations and subsequently forming implementation intentions might have also automatically triggered another behaviour change technique, known as ‘focus on past successes’ (Michie et al., 2013).

The results suggest that having read the recommendations and having formulated an implementation intention inspires older adults to think of its enactment. They may then realize that it is not always possible to enact the intentions as planned. Identified barriers for the enactment of their planned physical activity included the weather, Covid‐19 restrictions, commitments in other life domains or health issues. Interestingly, many older adults thought about possible barriers to their implementation intention without instructions to do so. This suggests that older adults may be inherently good at anticipating possible barriers to a behaviour, possibly showing their learning history. Results from the CH‐sample moreover suggest that older adults also spontaneously create plans to overcome the identified barriers, that is they used the behaviour change technique ‘problem solving’ (coping planning; Michie et al., 2013; Sniehotta et al., 2005), even though they were not prompted to do so by the interviewer. This is in line with research demonstrating that physical activity goal setting and use of plans to overcome barriers to physical activity increase with age (Anderson‐Bill et al., 2011; Ziegelmann et al., 2006).

This finding is encouraging, as a meta‐analysis has also shown that spontaneous planning has medium to large effects on physical activity behaviour (Carraro & Gaudreau, 2013). Also the use of problem solving is already known to enhance the effects of implementation intentions (Kwasnicka et al., 2013). However, this phenomenon was only seen in the CH‐sample where the participants were part of a more complex intervention study. They may have been more inclined to enact their physical activity plans. This could explain why in some intervention studies (Warner et al., 2016) healthy older adults were sceptical about implementation intentions without having the opportunity to set up coping plans at the same time.

### Strengths and limitations of the study

The findings of this study provide better understanding of what older adults think while planning their physical activity using implementation intentions. Since qualitative data from three different countries were analysed, this gives an insight into the views of older adults across a range of geographical and cultural contexts. Beliefs about implementation intentions encountered in all three sites are likely to be found in other samples. Other beliefs were found to be sample‐specific.

The self‐reflection on current physical activity only occurred in the UK‐sample, whereas unprompted coping plans were only formulated in the CH‐sample, even if since they had no instructions to think aloud which could have led to less detailed responses.

The CH‐sample may have been more inclined to implement their plans since they took part in an intervention study. They were also monitored with an accelerometer. In turn, the participants in the United Kingdom and Germany were not followed‐up. Indeed, there is evidence that wearing an accelerometer can boost motivation to implement self‐set goals (Mercer et al., 2016), and lead to more physical activity (Cadmus‐Bertram et al., 2015). Overall, these differences in procedures could have led to a systematic difference in their perception of the task. However, it is also noteworthy that most themes were found in all samples in spite of differences in procedures.

This suggests that the circumstances can influence the thought process of older adults when faced with an implementation intention task, which should not be neglected when using implementation intentions in an intervention. Yet, it is unclear if these differences in the samples are due to the geographic location, culture or other circumstances. For example, the data from the DE‐sample was assessed during the first wave of the Covid‐19 pandemic. Due closed places to exercise indoors (e.g. swimming pools, gyms) and governmental physical distancing restrictions limiting the plannability of physical activity the German data is not comparable to the other two samples. However, participants were aware of the remaining options and adapted their implementation intentions accordingly.

Also, it is important to note that some themes (i.e. the lack of necessity to plan and that planning triggers self‐reflection about physical activity) can also be solely caused by reading the WHO recommendations about physical activity as mentioned in the discussion. Indeed, giving information can be viewed as separate behaviour change technique. In regards to this, it is also to mention that this study only targeted moderate to vigorous physical activity mentioned in the recommendations. Therefore, the results could differ if we had invited older adults to choose their own intensity of physical activity instead of having them read the recommendations. Nevertheless, there is also some evidence that solely reading recommendations also can fail to change behaviour, but might boost awareness or the intention to be more active and therefrom set the stage for meaningful plans to increase it (Warburton & Bredin, 2017). Thus, the results apply only for implementation intentions when used with the WHO recommendations and targeting moderate to vigorous physical activity. When targeting other physical activities like walking or light activities around the house, older adults may experience less difficulties while planning. For example, such activities may be easier to implement every day or are influenced less by external circumstances. This could explain why especially older adults prefer slower paced activities (Alley et al., 2018).

As a caveat, participants from all samples were already quite active, motivated to participate in the study, and had a good understanding of their physical activity routines. Therefore, it is likely that they already knew their personal barriers and that some themes like ‘the lack of necessity to plan’ and to a lesser extent that ‘planning feels too restrictive’ can be specific to older adults, who are already active. Our findings should therefore be interpreted with caution and further research is warranted using the same implementation intention task and target behaviour with less active participants and older adults in different settings that might profit even more from learning how to use implementation intentions as self‐regulatory strategy (e.g. cardiac rehabilitation or retirement homes) (Luszczynska, 2006). Notably, since their necessity to have strategies to plan their physical activity is more pronounced, for example for reducing their risk of non‐communicable disease and associated morbidity (Geidl et al., 2020). In addition, in all three samples were higher educated than the general population, possibly even underreporting barriers to formulating implementation intentions (Allan et al., 2013).

There was no conclusive indication in the data whether older adults in our samples could be categorized into habitual planners (who like the planning task) and non‐planners (who oppose planning), which could be a helpful distinction to be explored as a possible moderator in future intervention studies. Nevertheless, this study gives an insight of the range of perspectives that may help explain why implementation intentions work really well with some people in some contexts, and less so in others.

### Practical implications

The results of this study encourage considering needs and resources of older adults when using implementation intentions to promote physical activity. Health practitioners should assess these (e.g. current physical activity, beliefs and attitudes towards planning), to then provide a tailored approach as to whether implementation intentions may be useful for certain older adults. From our findings, implementation intentions may only be accepted by (and possibly only be effective for) older adults who are not yet habitually physically active, have daily routines that can be used as cues, and do not feel restricted by planning their activities in such a format. For those who face one of these barriers that emerged in our interviews, different instructions for the planning task might be needed (e.g. use of more flexible cues such as good weather or mood), or different behaviour change techniques might be more suitable (such as coping planning). Also, the wording of the task could have an effect on how participants perceive it. Arguably, changing the focus from ‘planning’ to ‘implementing a new habit’, ‘fostering daily activity’ or ‘sitting less’, could trigger less reactance in older adults opposed to the concept of planning with implementation intentions.

## CONCLUSIONS

This study extends our knowledge on older adults' thoughts about implementation intentions related to physical activity, possible barriers and facilitators. These factors should be considered when designing future interventions using implementation intentions for older adults. Tailoring an intervention for a certain population, like older adults, can enhance its effectiveness and sustainability (Barker, 2018; Gellert et al., 2014). Also, older adults should be involved in planning and evaluating behaviour change interventions (Kok, 2018). This is in line with Hankonen (2018), who states that behaviour change techniques can only work, if participants understand how to use the strategies, acknowledge their usefulness, and thereby actively engage with the learned strategy in their daily life. Therefore, understanding the thought processes of older adults when forming implementation intentions hopefully helps researchers and practitioners to reflect *who* the agents of the behaviour change techniques are and adapt them according to their needs (Hankonen, 2018).

## AUTHOR CONTRIBUTIONS

**Valérie Désirée Bösch:** Formal analysis; writing – original draft; writing – review and editing. **Lisa Marie Warner:** Conceptualization; investigation; methodology; project administration; resources; supervision; validation; writing – review and editing. **Samuel R. Nyman:** Conceptualization; investigation; methodology; project administration; resources; supervision; validation; writing – review and editing. **Julius Haftenberger:** Conceptualization; data curation; investigation; writing – review and editing. **Kye Clarke:** Conceptualization; data curation; investigation; writing – review and editing. **Jennifer Inauen:** Conceptualization; investigation; methodology; project administration; resources; supervision; validation; writing – original draft; writing – review and editing.

## CONFLICT OF INTEREST

All authors declare that they have no conflicts of interest.

## Supporting information


Appendix S1.

## Data Availability

The data that support the findings of this study are available on request from the corresponding author. The data are not publicly available due to privacy or ethical restrictions.
