# Supplementary material for: What do older adults think about when formulating implementation intentions for physical activity? Evidence from a qualitative study
Source: Br J Health Psychol. 2022 Aug 24;28(1):221–36. doi: 10.1111/bjhp.12621 (PMC10087560; doi:10.1111/bjhp.12621)
Supplement: Supplementary file 1 — Appendix S1. [file BJHP-28-221-s001.docx]

**Supporting Information**

All mentioned Supplements and supporting information can be found in the Open Science Framework under following link:

<https://osf.io/gu9d8/?view_only=2a19fddc8d574a7caa0dd9bdd8318680>
